# Analysis of risk factors for early recurrence after radiofrequency ablation in patients with atrial fibrillation and construction of a nomogram predictive model

**DOI:** 10.3389/fcvm.2026.1659637

**Published:** 2026-02-17

**Authors:** Jia-Nan Wang, Hui-Lan Liu, Hui-Hong Hong, Ting-Pei Zhuang, Bing Wu

**Affiliations:** 1 Department of Cardiology, Quanzhou First Hospital Affiliated to Fujian Medical University, Quanzhou City, Fujian Province, China; 2 Department of Cardiology, Quanzhou First Hospital, Quanzhou City, Fujian Province, China

**Keywords:** atrial fibrillation, early recurrence, nomogram, obstructive sleep apnea syndrome, radiofrequency catheter ablation

## Abstract

**Background:**

Early recurrence of atrial fibrillation (AF) after radiofrequency catheter ablation remains common and undermines procedural success. This study aimed to identify clinical, echocardiographic, and biochemical predictors of early atrial tachyarrhythmia recurrence and to construct a nomogram for individualized risk assessment.

**Methods:**

This retrospective cohort study included 276 consecutive patients with AF undergoing first-time radiofrequency catheter ablation between January 2021 and December 2024. Early recurrence was defined as any documented AF, atrial flutter, or atrial tachycardia lasting ≥30 s within 3 months post-procedure. All patients received short-term oral amiodarone for 3 months to cover the 90-day blanking period. Multivariable logistic regression was used to identify independent predictors. A nomogram was developed using the rms package in R. Discrimination was assessed using the area under the receiver operating characteristic curve (AUC). Internal validation and calibration were assessed using bootstrap resampling (1,000 iterations) with an optimism-corrected concordance index (C-index) and calibration plots, and clinical utility was evaluated using decision curve analysis (DCA).

**Results:**

Univariate analysis showed higher body mass index (BMI), larger left atrial diameter (LAD), higher B-type natriuretic peptide (BNP) levels, and higher prevalences of dyslipidemia, obstructive sleep apnea syndrome (OSAS), and persistent AF in patients with early recurrence (all *p* < 0.05). Multivariable analysis identified BMI (per 1 kg/m^2^; OR 1.753, 95% CI 1.443–2.113; *p* < 0.001), LAD (per 5 mm; OR 1.556, 95% CI 1.152–2.102; *p* = 0.004), BNP (per 100 ng/L; OR 1.703, 95% CI 1.373–2.053; *p* < 0.001), persistent AF (OR 4.203, 95% CI 1.324–13.507; *p* = 0.017), and OSAS (OR 3.405, 95% CI 1.081–11.005; *p* = 0.041) as independent predictors. The nomogram showed acceptable discrimination (AUC 0.761, 95% CI 0.693–0.851), stable internal validation (optimism-corrected C-index 0.758), good calibration, and favorable net clinical benefit on DCA.

**Conclusions:**

Elevated BMI, enlarged LAD, increased BNP, persistent AF, and OSAS independently predict early atrial tachyarrhythmia recurrence after ablation. This nomogram may support individualized early post-ablation risk stratification, pending external validation in multicenter cohorts.

## 1 Introduction

Atrial fibrillation (AF) is the most common sustained arrhythmia encountered in clinical practice, affecting an estimated 2%–4% of the adult population and conferring a substantially increased risk of stroke, heart failure, and all-cause mortality (1). The structural and electrophysiological remodeling underlying AF, characterized by atrial dilatation, interstitial fibrosis, and ion channel dysregulation, promotes the initiation and maintenance of disorganized atrial activation, resulting in impaired hemodynamics and an increased propensity for thromboembolic events. Catheter radiofrequency ablation (RFA) has become a cornerstone of interventional AF management and has demonstrated superiority over antiarrhythmic drug therapy in achieving freedom from atrial arrhythmia and improving quality of life (2, 3). Blanking period arrhythmia refers to atrial tachyarrhythmia episodes occurring during the early post-ablation blanking period, traditionally defined as the first 90 days after AF catheter ablation (4, 5). These events include atrial fibrillation, atrial flutter, or atrial tachycardia lasting at least 30 s. Previous studies indicate that atrial arrhythmias occur in approximately 20%–50% of patients during this early post-ablation phase; however, only a subset subsequently develop late recurrence, highlighting that early recurrence is a heterogeneous phenomenon rather than a uniform marker of ablation failure (6, 7). These arrhythmias are commonly driven by transient and potentially reversible mechanisms, such as ablation-related inflammation, autonomic imbalance, short-term electrical remodeling, and incomplete lesion maturation prior to stable scar formation (8). Therefore, arrhythmias during the blanking period do not invariably represent durable procedural failure. Nevertheless, blanking period arrhythmias are clinically relevant rather than benign (9). They occur in a substantial proportion of patients and provide prognostic information, particularly when considered in a time-dependent manner (5). Emerging evidence supports stratifying early recurrences into very early recurrence (for example, within 7 days or up to 30 days), which is more often linked to acute peri-procedural perturbations and is less predictive of late failure, vs. later blanking period recurrence (approximately 1–3 months), which more strongly reflects pulmonary vein reconnection and adverse atrial substrate remodeling and is associated with subsequent late recurrence (10, 11). Clinically, blanking period arrhythmia can be viewed as an early stress test of the post-ablation atrial substrate and may help identify patients who warrant intensified rhythm surveillance and risk-factor optimization (12–14).

Several clinical and procedural variables have been associated with early recurrence of atrial fibrillation (ERAF). Advanced age and longer AF duration before ablation are linked to higher risk, likely reflecting more extensive atrial substrate remodeling (15). Comorbidities such as hypertension, diabetes mellitus, and obesity may further aggravate atrial remodeling through systemic inflammation and oxidative stress. In addition, procedural factors, including inadequate lesion delivery and incomplete pulmonary vein isolation, may increase the likelihood of pulmonary vein reconnection and persistence of arrhythmogenic triggers (16). Although multivariable regression analyses have identified individual predictors, translation of these findings into individualized risk estimation remains limited. Nomograms provide a graphical representation of a predictive model that enables patient-specific risk assessment by integrating multiple heterogeneous variables into a single tool. Such models have demonstrated utility in oncology and cardiovascular medicine, often offering improved discrimination and calibration compared with conventional risk scores (17).

In this study, we retrospectively analyzed a cohort of patients undergoing first-time radiofrequency catheter ablation for AF to identify independent predictors of early arrhythmia recurrence. We then constructed and internally validated a nomogram incorporating clinical, echocardiographic, and biochemical variables. Our aim was to develop a practical prognostic tool with reliable discrimination and calibration to support personalized post-ablation management and potentially improve subsequent rhythm outcomes.

## 2 Methods

### 2.1 Study design

**Figure 1:**
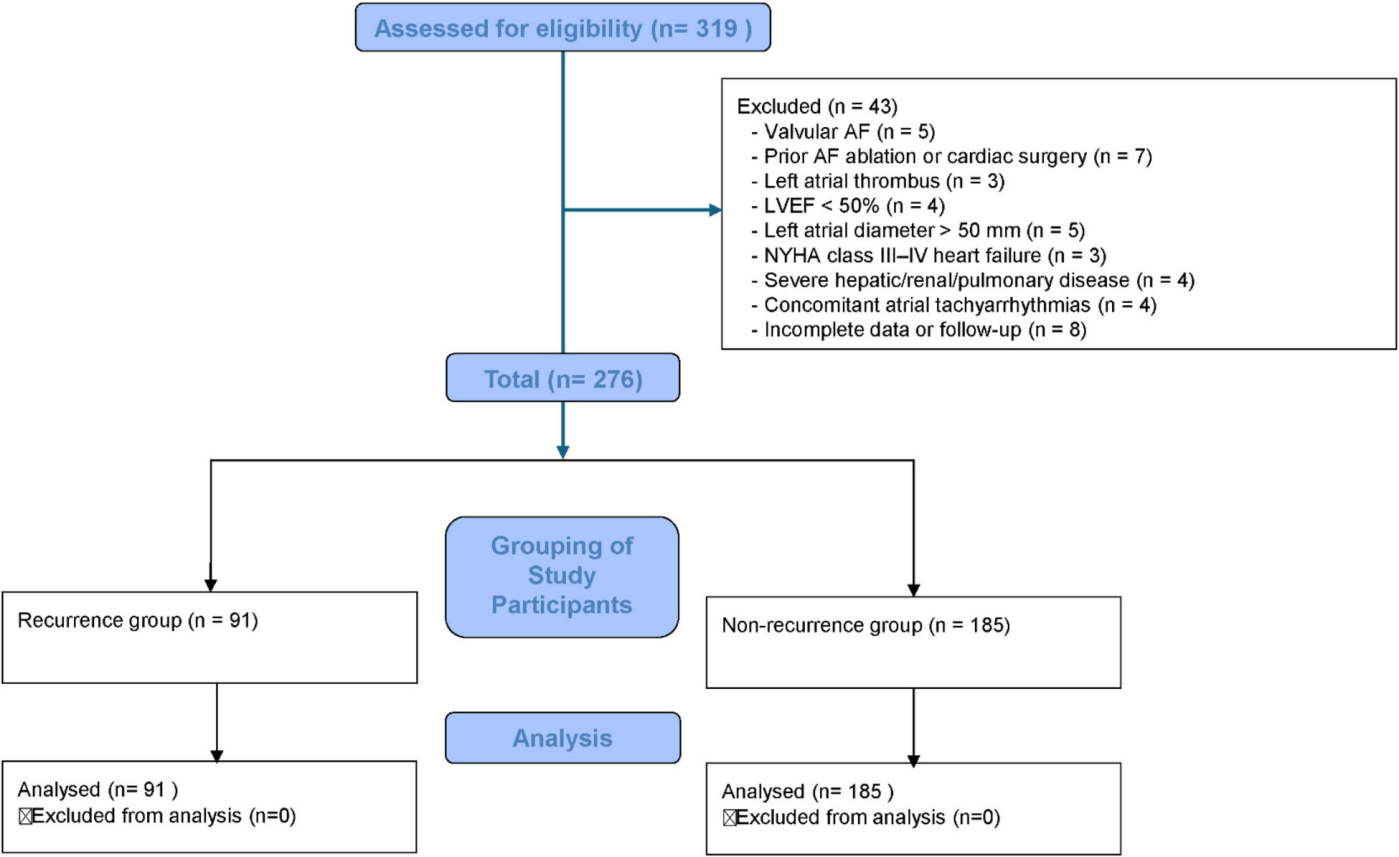
Flowchart of patient selection and grouping.

This retrospective cohort study enrolled 276 patients with atrial fibrillation who underwent radiofrequency ablation at our institution between January 2021 and December 2024, all of whom met the predefined inclusion and exclusion criteria. Patients were stratified according to the occurrence of atrial tachyarrhythmia recurrence within three months post-procedure into a recurrence group (*n* = 91) and a non-recurrence group (*n* = 185) (Figure 1). The development and reporting of this prediction model adhered to the TRIPOD (Transparent Reporting of a multivariable prediction model for Individual Prognosis Or Diagnosis) guidelines (18). The study was reviewed and approved by the hospital's ethics committee. All procedures adhered to relevant guidelines and the principles of the Declaration of Helsinki. Participant data were anonymized prior to analysis to ensure confidentiality and privacy.

### 2.2 Inclusion and exclusion criteria

#### 2.2.1 Inclusion criteria

1. Atrial fibrillation confirmed by 12-lead electrocardiography, defined by absence of discrete P waves, presence of fibrillatory (f) waves of varying amplitude and frequency, and absolutely irregular RR intervals.
2. Age ≥18 years at the time of ablation.
3. Indication for radiofrequency catheter ablation according to established guidelines, with no contraindications.
4. Left ventricular ejection fraction ≥50 % and left atrial anteroposterior diameter ≤50 mm on transthoracic echocardiography.
5. Complete availability of clinical, procedural, and follow-up data.

#### 2.2.2 Exclusion criteria

1. Valvular atrial fibrillation (including moderate-to-severe mitral stenosis or presence of mechanical heart valves).
2. Prior catheter ablation for atrial fibrillation or history of cardiac surgery.
3. Left atrial thrombus detected on pre-procedural transesophageal echocardiography.
4. New York Heart Association class III–IV heart failure.
5. Severe hepatic (Child–Pugh class C), renal (eGFR < 30 mL/min/1.73 m^2^), or pulmonary (GOLD stage III–IV) dysfunction.
6. Concomitant atrial tachyarrhythmias requiring alternative ablation strategies.

### 2.3 Diagnostic criteria for early recurrence of atrial fibrillation

ERAF was defined as any documented episode of atrial fibrillation, atrial flutter, or atrial tachycardia lasting at least 30 s and occurring within the three-month post-ablation blanking period. Episodes were considered ERAF only if confirmed by a standard 12-lead electrocardiogram, at least 24-hour Holter monitoring, a patient-activated event recorder, or an implantable loop recorder, regardless of symptom status. All recordings were independently adjudicated by two electrophysiologists to ensure objective confirmation of atrial tachyarrhythmia recurrence.

### 2.4 Operative technique

Prior to ablation, all patients underwent a comprehensive preprocedural evaluation, including 12-lead electrocardiography, transthoracic echocardiography, and contrast-enhanced cardiac computed tomography with three-dimensional reconstruction to assess cardiac function and delineate pulmonary vein anatomy. Under continuous electrocardiographic monitoring and after local infiltration with 1% lidocaine, patients were placed in the supine position and right femoral venous access was obtained. A Swartz sheath was advanced into the left atrium via transseptal puncture under fluoroscopic guidance, followed by selective pulmonary vein angiography to identify the pulmonary vein ostia. Using a three-dimensional electroanatomic mapping system (e.g., CARTO or EnSite) and a single-use mapping catheter, circumferential pulmonary vein isolation was performed with an open-irrigated radiofrequency ablation catheter irrigated with 0.9% saline. When fragmented electrograms were identified at the antrum, additional targeted ablation was applied until electrogram fragmentation was eliminated. Procedural endpoints included restoration of stable sinus rhythm and noninducibility of atrial fibrillation during programmed stimulation. Postoperatively, all patients received oral amiodarone for three months to maintain rhythm stability to cover the three months post-ablation blanking period (19–22).

### 2.5 Clinical data collection

Demographic and baseline clinical characteristics were obtained from medical records and included sex, age, body mass index, history of tobacco and alcohol use, and comorbid conditions, including hypertension, diabetes mellitus, dyslipidemia, and obstructive sleep apnea syndrome. Disease-specific variables included the duration of atrial fibrillation and arrhythmia subtype, categorized as paroxysmal or persistent based on electrocardiographic documentation. Left atrial diameter was measured using transthoracic echocardiography (23–25). Preprocedural laboratory assessments, performed within seven days prior to ablation, included serum albumin, alanine aminotransferase, aspartate aminotransferase, creatinine, blood urea nitrogen, uric acid, and B-type natriuretic peptide.

### 2.6 Statistical analysis

All statistical analyses were performed using SPSS version 28.0 (IBM Corp., Armonk, NY, USA) and R software version 4.3.3 (R Foundation for Statistical Computing, Vienna, Austria). Categorical variables are presented as counts and percentages and were compared using the chi-square test. Continuous variables are expressed as mean plus or minus standard deviation and were analyzed using two-sided independent-samples t tests. Independent risk factors for early atrial arrhythmia recurrence following radiofrequency ablation were identified using multivariable binary logistic regression. Multicollinearity among candidate predictors was assessed prior to multivariable modeling using variance inflation factors (VIFs). A VIF value <5 was considered indicative of no clinically meaningful multicollinearity. To reduce the risk of overfitting, model complexity was controlled by limiting the number of predictors included in the final multivariable model. Adequacy of sample size for multivariable modeling was assessed using the events-per-variable (EPV) principle. A nomogram-based predictive model was subsequently developed using the rms package in R. Model discrimination was assessed using the area under the receiver operating characteristic curve (AUC). Internal validation was performed using bootstrap resampling with 1,000 iterations to estimate optimism-corrected discrimination, expressed as the concordance index (C-index), and to generate calibration plots. Decision curve analysis (DCA) was applied to evaluate the clinical utility of the nomogram. All statistical tests were two-tailed, and a *p* value <0.05 was considered statistically significant.

## 3 Results

### 3.1 Univariable analysis of clinical and laboratory parameters

Univariable comparisons between patients with and without early recurrence following radiofrequency ablation are summarized in Table 1. Demographic characteristics, including age, sex distribution, smoking status, alcohol consumption, hypertension, and diabetes mellitus, were comparable between the two groups, with no statistically significant differences observed (all *p* > 0.05). In contrast, patients in the recurrence group exhibited a significantly higher mean body mass index and a greater prevalence of dyslipidemia and obstructive sleep apnea syndrome compared with those without recurrence (BMI: 27 ± 3 vs. 23 ± 3 kg/m^2^, *p* < 0.001; dyslipidemia: 50.5% vs. 29.2%, *p* = 0.001; OSAS: 45.1% vs. 24.9%, *p* = 0.001). Persistent atrial fibrillation was also more frequent among patients with recurrence than among those without recurrence (35.2% vs. 17.8%, *p* = 0.001). Echocardiographic and laboratory parameters further differentiated the two groups. The recurrence cohort demonstrated a significantly larger left atrial diameter (45 ± 4 mm vs. 39 ± 4 mm, *p* < 0.001) and lower serum albumin levels (34 ± 5 g/L vs. 37 ± 4 g/L, *p* < 0.001). Although most liver enzymes were similar between groups, alanine aminotransferase levels were modestly lower in patients with recurrence (58 ± 13 U/L vs. 62 ± 14 U/L, *p* = 0.023). In addition, B-type natriuretic peptide concentrations were significantly higher in the recurrence group (520 ± 151 ng/L vs. 450 ± 151 ng/L, *p* < 0.001). No significant differences were observed between groups in aspartate aminotransferase, creatinine, blood urea nitrogen, or uric acid levels.

**Table 1:** Univariable comparison of clinical and laboratory parameters between patients With and without early recurrence.

Variable	Non-recurrence (*n* = 185)	Recurrence (*n* = 91)	*Χ*^2^/*t*	*P*-value
Demographics and Comorbidities				
Male, *n* (%)	106 (57.3)	55 (60.4)	0.135	0.713
Age (years), mean ± SD	60 ± 12	60 ± 11	0.001	0.999
BMI (kg/m^2^), mean ± SD	23 ± 3	27 ± 3	−10.413	<0.001
Smoking history, *n* (%)	49 (26.5)	25 (27.5)	0.001	0.977
Alcohol use, *n* (%)	61 (33.0)	35 (38.5)	0.586	0.444
Hypertension, *n* (%)	71 (38.4)	33 (36.3)	0.044	0.835
Diabetes mellitus, *n* (%)	61 (33.0)	40 (44.0)	2.715	0.099
Dyslipidemia, *n* (%)	54 (29.2)	46 (50.5)	12.05	0.001
OSAS, *n* (%)	46 (24.9)	41 (45.1)	11.52	0.001
AF duration (years), mean ± SD	2.3 ± 0.8	2.3 ± 0.7	0.001	0.999
Arrhythmia Subtype			10.17	0.001
Persistent AF, *n* (%)	33 (17.8)	32 (35.2)		
Paroxysmal AF, *n* (%)	152 (82.2)	59 (64.8)		
Echocardiographic and Laboratory Data				
LAD (mm), mean ± SD	39 ± 4	45 ± 4	−11.72	<0.001
Albumin (g/L), mean ± SD	37 ± 4	34 ± 5	5.381	<0.001
ALT (U/L), mean ± SD	62 ± 14	58 ± 13	2.284	0.023
AST (U/L), mean ± SD	45 ± 13	48 ± 11	−1.893	0.059
Creatinine (*μ*mol/L), mean ± SD	68 ± 10	70 ± 9	−1.613	0.108
BUN (mmol/L), mean ± SD	11 ± 4	11 ± 5	0.001	0.999
Uric acid (μmol/L), mean ± SD	360 ± 90	350 ± 90	0.868	0.386
BNP (ng/L), mean ± SD	450 ± 151	520 ± 151	−3.621	<0.001

BMI, body mass index; OSAS, obstructive sleep apnea syndrome; AF, atrial fibrillation; LAD, left atrial diameter; ALT, alanine aminotransferase; AST, aspartate aminotransferase; BUN, blood urea nitrogen; BNP, B-type natriuretic peptide.

### 3.2 Independent predictors of early recurrence

In multivariable logistic regression analysis (Table 2), several variables emerged as independent predictors of early atrial tachyarrhythmia recurrence following radiofrequency ablation. After adjustment for potential confounders, each 1 kg/m^2^ increment in body mass index conferred a 75% increase in recurrence odds (OR 1.753, 95% CI 1.443–2.113; *β* 0.561; *p* < 0.001). Similarly, each 5-mm increase in left atrial diameter was associated with a significant elevation in recurrence risk (OR 1.556, 95% CI 1.152–2.102; *β* 0.442; *p* = 0.004). Elevated B-type natriuretic peptide also independently predicted recurrence, with each 100 ng/L increase associated with a 70% increase in the odds of early recurrence (OR 1.703, 95% CI 1.373–2.053; *β* 0.532; *p* < 0.001). Regarding comorbidities and arrhythmia subtype, patients with persistent AF exhibited over fourfold greater odds of early recurrence compared to those with paroxysmal AF (OR 4.203, 95% CI 1.324–13.507; *β* 1.438; *p* = 0.017). The presence of obstructive sleep apnea syndrome similarly increased recurrence risk more than threefold (OR 3.405, 95% CI 1.081–11.005; *β* 1.227; *p* = 0.041). In contrast, dyslipidemia did not retain significance in the adjusted model (OR 1.783, 95% CI 0.632–5.204; *β* 0.578; *p* = 0.283). For continuous predictors, effect estimates are reported per the scaled increments specified in Table 2 to facilitate clinical interpretability. Assessment of multicollinearity demonstrated no substantial correlation among predictors included in the final multivariable model. Variance inflation factor values ranged from 1.3 to 2.9, with all VIFs <5, indicating the absence of concerning multicollinearity.

**Table 2:** Multivariable logistic regression analysis of early recurrence risk factors.

Variable	*β* Value	Standard Error	Wald Value	OR	95% CI	*P*-value
Body mass index (per 1 kg/m^2^ increase)	0.561	0.097	33.223	1.753	1.443–2.113	<0.001
Left atrial diameter (per 5 mm increase)	0.442	0.153	8.300	1.556	1.152–2.102	0.004
B-type natriuretic peptide (per 100 ng/L increase)	0.532	0.103	26.881	1.703	1.373–2.053	<0.001
Persistent AF (vs. paroxysmal AF)	1.438	0.593	5.875	4.203	1.324–13.507	0.017
Obstructive sleep apnea syndrome (yes vs. no)	1.227	0.593	4.267	3.405	1.081–11.005	0.041
Dyslipidemia (yes vs. no)	0.578	0.539	1.148	1.783	0.632–5.204	0.283
Intercept (constant)	−0.71	0.25	8.1	—	—	0.004

AF, atrial fibrillation; OR, odds ratio; CI, confidence interval; *β*, regression coefficient.

The intercept represents the baseline log-odds of early recurrence when all predictors are zero.

### 3.3 Nomogram construction and discriminative performance

**Figure 2:**
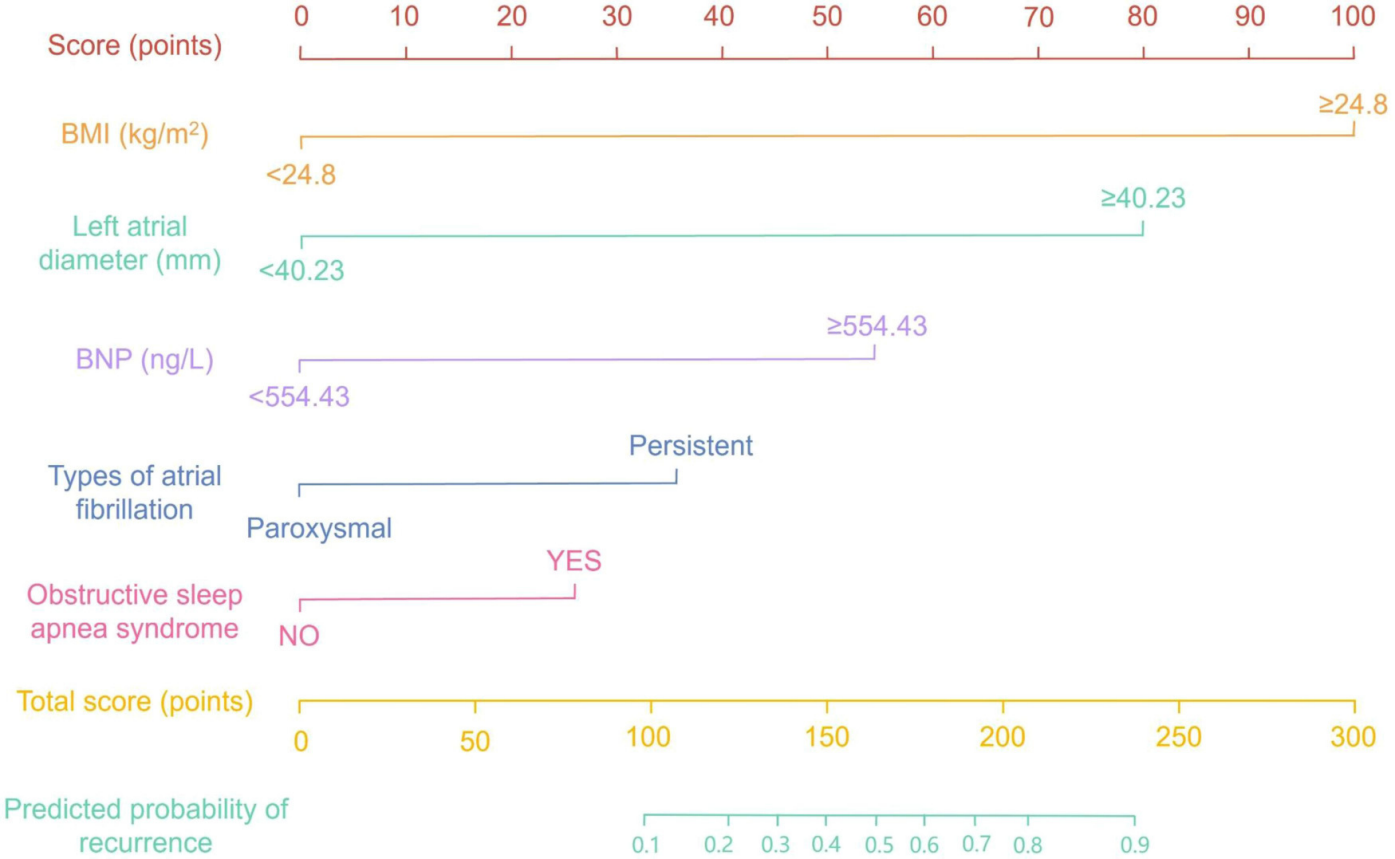
Nomogram for predicting the risk of early atrial fibrillation recurrence within three months after radiofrequency catheter ablation.

A risk-prediction nomogram was developed using the five variables independently associated with early atrial arrhythmia recurrence on multivariable logistic regression: body mass index, left atrial diameter, B-type natriuretic peptide, persistent vs. paroxysmal AF, and obstructive sleep apnea syndrome (Figure 2). Each predictor was assigned a point value proportional to its regression coefficient; summing these yields a total score corresponding to an individual's probability of early recurrence.

**Figure 3:**
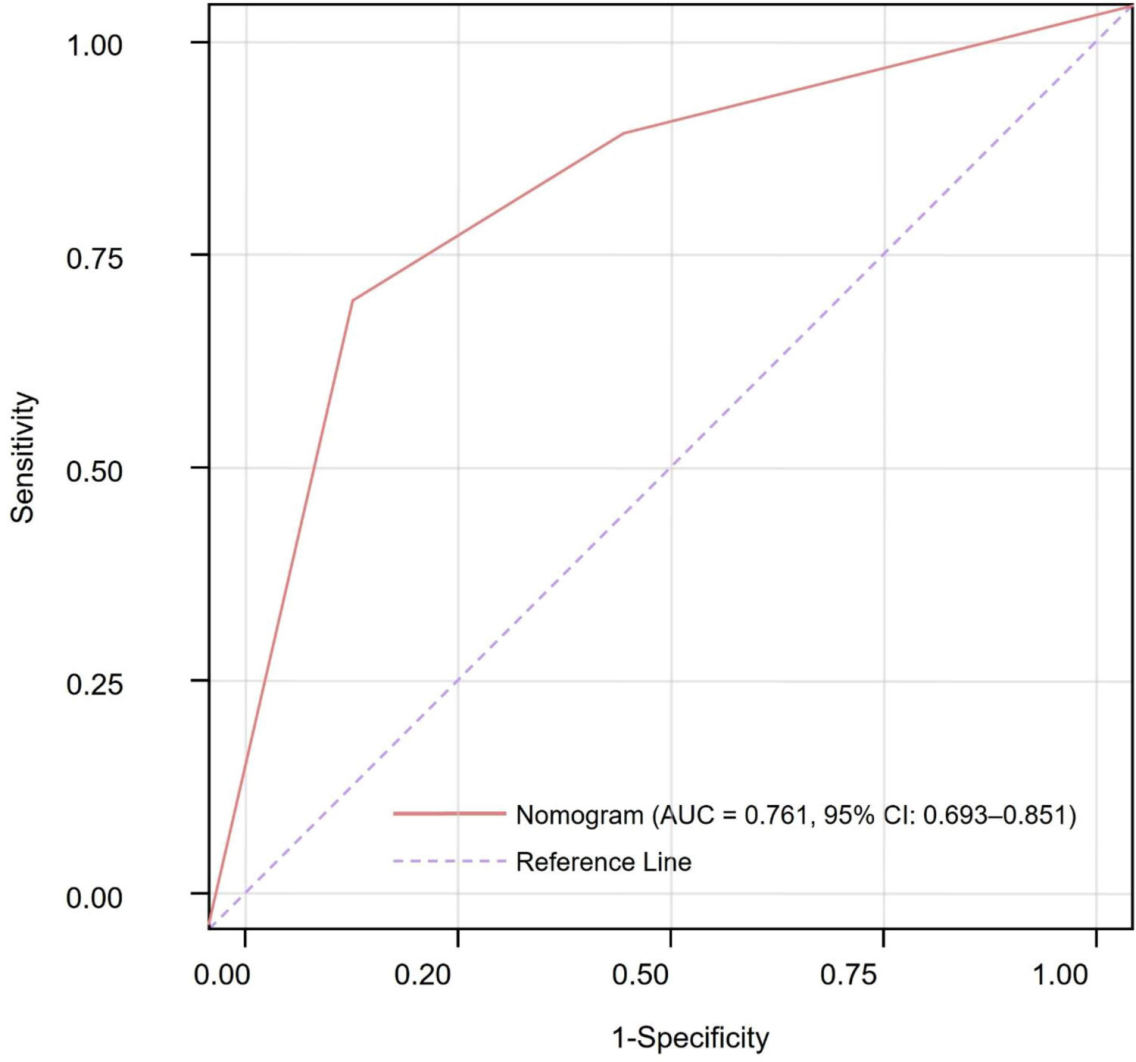
Receiver operating characteristic curve assessing the discriminative performance of the nomogram for early post-ablation atrial fibrillation recurrence.

The model's discriminative performance was evaluated using receiver operating characteristic analysis. The nomogram achieved an AUC of 0.761 (95% CI, 0.693–0.851). At the optimal cutoff determined by the Youden index, sensitivity was 69.3% and specificity was 75.7% (Figure 3), indicating acceptable discrimination.

### 3.4 Calibration and clinical utility

**Figure 4:**
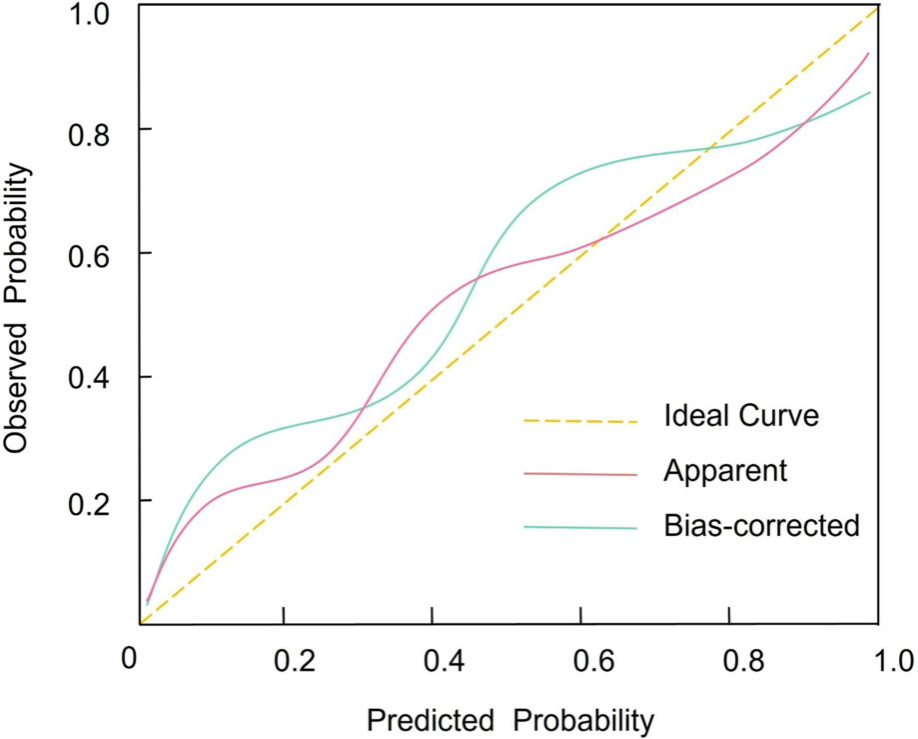
Calibration plot comparing predicted versus observed probabilities of early atrial fibrillation recurrence following radiofrequency ablation.

**Figure 5:**
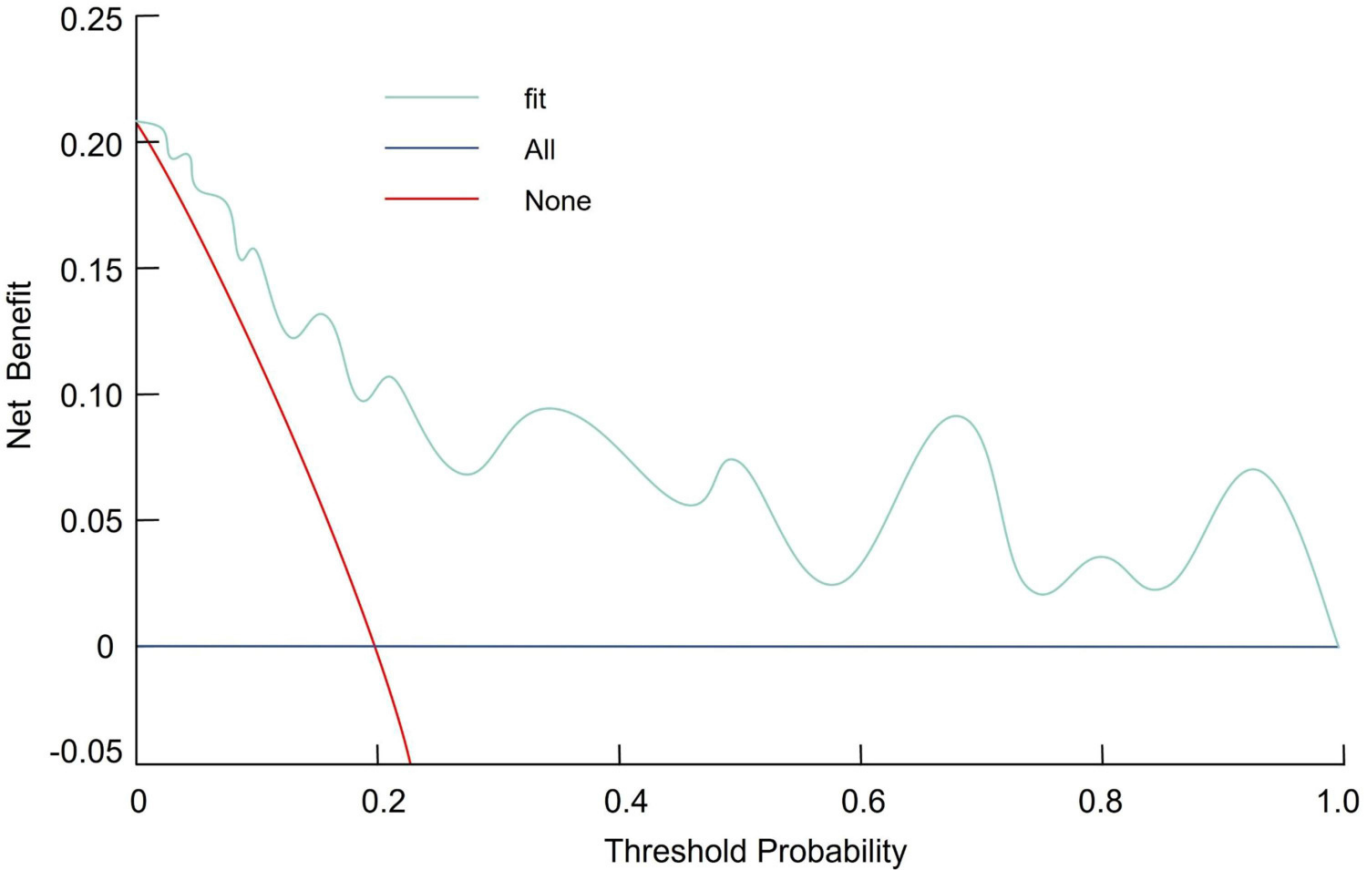
Decision curve analysis illustrating the net clinical benefit of using the nomogram to guide management decisions for early recurrence risk after ablation.

Internal validation was performed using bootstrap resampling (1,000 iterations). Bootstrap validation yielded an optimism-corrected AUC (C-index) of 0.758, which was very close to the apparent AUC of 0.761, indicating minimal optimism and limited overfitting. Calibration was assessed by the Hosmer–Lemeshow goodness-of-fit test (*χ*^2^ = 2.369, *p* = 0.912) and calibration plots, which demonstrated good agreement between predicted and observed recurrence risks across deciles (Figure 4). DCA showed that the nomogram provided a higher net benefit than the treat-all or treat-none strategies across a range of clinically relevant threshold probabilities, supporting its potential utility for individualized post-ablation management (Figure 5). Early recurrence occurred in 91 patients. With five predictors retained in the final multivariable model, the EPV was 18.2, supporting adequate model stability and a low risk of overfitting. These findings are consistent with the results of bootstrap internal validation, which demonstrated minimal optimism.

### 3.5 *Post Hoc* power analysis

A *post hoc* weighted power analysis was conducted to assess the overall statistical power of the multivariable logistic regression model. Power for each independent predictor was estimated based on the corresponding Wald statistic (*β*/SE), assuming a two-sided significance level of *α* = 0.05. To provide an overall measure of model adequacy, equal weights (20%) were assigned to each of the five independent predictors included in the final model (body mass index, left atrial diameter, B-type natriuretic peptide, atrial fibrillation subtype, and obstructive sleep apnea syndrome). The resulting weighted average statistical power was 80.9%, exceeding the conventional threshold of 80% and indicating that the model was adequately powered to detect the observed associations.

## 4 Discussion

In this cohort of 276 patients undergoing first-time catheter radiofrequency ablation for AF, we observed that higher BMI, enlarged LAD, elevated BNP, persistent AF subtype, and coexisting OSAS each independently predicted early arrhythmia recurrence within three months of the procedure. In contrast, dyslipidemia, although more prevalent in those with recurrence on univariable analysis, did not retain significance after adjustment for these stronger predictors. These findings underscore that both structural cardiac changes (LAD), biomarkers of myocardial stress (BNP), AF chronicity (persistent vs. paroxysmal), systemic comorbidity (OSAS), and metabolic factors (BMI) collectively shape the post-ablation substrate that predisposes to AF relapse. This study offers several novel and clinically relevant contributions. First, it specifically addresses early atrial tachyarrhythmia recurrence during the post-ablation blanking period, an underexplored yet clinically important phase that reflects early atrial vulnerability and may influence subsequent rhythm outcomes. Second, by integrating readily available clinical, echocardiographic, and biochemical variables, we identified a concise set of independent predictors and translated them into a pragmatic nomogram for individualized risk estimation. Unlike studies focusing on isolated predictors, our multivariable framework captures the combined effects of metabolic burden, structural remodeling, neurohumoral stress, arrhythmia chronicity, and systemic comorbidity. The model demonstrated acceptable discrimination, stable internal validation, and potential clinical utility. Clinically, this tool may support risk-adapted rhythm surveillance, tailored follow-up strategies, and individualized management planning during the blanking period, thereby facilitating more efficient and personalized post-ablation care.

Mechanistically, elevated BMI reflects increased adiposity, which contributes to systemic inflammation, oxidative stress, and elevated cardiac workload. These factors accelerate atrial remodeling, characterized by interstitial fibrosis and myocyte hypertrophy, ultimately compromising the durability of electrical isolation achieved through ablation. Similarly, left atrial enlargement indicates chronic pressure and volume overload, resulting in a larger substrate of arrhythmogenic myocardium capable of sustaining focal or reentrant circuits despite pulmonary vein isolation. Elevated BNP serves as a surrogate marker for elevated intracardiac filling pressures and subclinical ventricular dysfunction; atrial stretch and wall tension promote electrophysiological heterogeneity, increasing susceptibility to arrhythmias (26, 27). Persistent AF represents a progression from episodic to sustained arrhythmia and is associated with more advanced electrical remodeling, including shortened refractory periods and atrial fibrosis. This pathological substrate is less responsive to pulmonary vein isolation alone compared to paroxysmal AF. Additionally, OSAS induces intermittent hypoxia and sympathetic activation, fostering atrial inflammation and fibrosis. Untreated OSAS impairs long-term maintenance of sinus rhythm following ablation. Collectively, these predictors underscore the multifactorial pathogenesis of early AF recurrence, involving mechanical, biochemical, electrophysiological, and systemic mechanisms, and identify potential targets for pre- and post-procedural optimization (28, 29).

Our identification of BMI, LAD, BNP, AF subtype, and OSAS as predictors of early recurrence aligns with and expands upon prior findings in the field. Obesity has consistently been associated with increased AF incidence and diminished ablation efficacy; clinical trials targeting weight reduction have demonstrated significant decreases in recurrence rates, supporting our conclusion that BMI is one of the most impactful modifiable risk factors. Likewise, left atrial enlargement has been validated in multiple large registries and meta-analyses as a strong marker of atrial substrate burden. Enlarged atria tend to contain greater degrees of fibrotic remodeling and are more likely to harbor non–pulmonary vein triggers, contributing to elevated relapse risk across diverse populations (30, 31). The use of BNP as a biomarker for predicting ablation outcomes is supported by previous studies showing that baseline natriuretic peptide levels are correlated with both short- and long-term procedural success. Although some investigations have utilized N-terminal pro-BNP instead of BNP, both markers reflect adverse hemodynamic load and myocardial stretch. Incorporating BNP into our predictive nomogram is consistent with these prior reports and highlights the emerging role of cardiac biomarkers in stratifying recurrence risk following catheter ablation (32, 33).

Persistent AF is widely acknowledged as a more complex substrate for ablation, with consistently lower success rates compared to paroxysmal AF across nearly all comparative studies (34, 35). Our finding that persistent AF independently increases the odds of early recurrence by more than fourfold aligns with this established trend. To address this challenge, many centers have adopted adjunctive lesion sets or hybrid strategies, recognizing that pulmonary vein isolation alone is frequently insufficient in this subgroup. The role of OSAS in ablation outcomes has gained increasing recognition, with accumulating evidence indicating that untreated sleep apnea significantly elevates recurrence risk. Studies have shown that continuous positive airway pressure therapy can mitigate this risk (36, 37). Our observation of a three- to fourfold increased risk of early recurrence in patients with OSAS highlights the critical importance of routine screening and treatment for sleep-disordered breathing, as endorsed by current consensus guidelines, although not yet universally implemented in clinical practice (38). Finally, the absence of an independent association between dyslipidemia and early recurrence in our cohort is consistent with the heterogeneous findings reported in the literature (1, 39–42). While statins may offer anti-inflammatory and pleiotropic benefits in AF, serum cholesterol levels alone have not reliably predicted ablation outcomes when more robust determinants, such as atrial size and metabolic syndrome components, are considered (43, 44).

The ATLAS score, developed by Mesquita et al. (42), was designed to estimate AF recurrence after first pulmonary vein isolation using a large two-center cohort with a mean follow-up of approximately 4.2 years. It incorporates demographic factors (age and sex), arrhythmia phenotype (non-paroxysmal AF), lifestyle exposure (current smoking), and indexed left atrial volume, and demonstrated good long-term discriminative performance (c-statistic ≈ 0.75). The ATLAS score is primarily intended for long-term risk stratification and pre-procedural patient selection, whereas early recurrence is more closely related to transient post-ablation mechanisms such as inflammation, atrial stunning, and autonomic imbalance, which are not explicitly captured by this model. The APPLE score, proposed by Kornej et al. (45), was developed to predict AF recurrence between 3 and 12 months after ablation and includes persistent AF, left atrial diameter, renal dysfunction, advanced age, and reduced left ventricular systolic function. Although APPLE showed better performance than CHADS₂ and CHA_2_DS_2_-VASc for mid-term rhythm outcomes, its discriminative ability remained modest (AUC ≈ 0.63). In addition, APPLE was not designed for use during the blanking period and does not account for factors potentially relevant to early recurrence, such as obstructive sleep apnea syndrome or biochemical markers of atrial wall stress. The MB-LATER score, developed by Mujović et al. (46), targets a distinct clinical context by predicting very late AF recurrence (>12 months) in patients who remained arrhythmia-free during the first post-ablation year. Early recurrence is incorporated as a component of this score, reflecting its role as a predictor of subsequent late relapse rather than as an outcome to be estimated. These differences indicate that individual models address different phases of the post-ablation course. ATLAS and APPLE are more applicable to pre-procedural assessment and mid- to long-term prognosis, whereas MB-LATER informs long-term surveillance. The present nomogram is specifically tailored to the early post-ablation blanking period and provides individualized risk estimation to support targeted monitoring and early post-procedural management.

This study has several notable strengths. Most importantly, a comprehensive multivariable framework was applied to integrate clinical, echocardiographic, biochemical, and comorbidity-related factors, which were subsequently translated into a practical and user-friendly nomogram. The model demonstrated acceptable discrimination (AUC 0.761), stable internal validation (optimism-corrected C-index 0.758), and net clinical benefit on DCA. Importantly, the nomogram is intended to support individualized post-ablation risk stratification rather than to imply short-term reversibility of structural or biochemical determinants (47, 48). By focusing specifically on the three-month blanking period, this study addresses a clinically relevant phase characterized by frequent atrial tachyarrhythmias and ongoing management flexibility, including intensified rhythm monitoring, tailored follow-up strategies, and individualized patient counseling (49). Despite uniform short-term amiodarone use, early recurrences persisted, highlighting residual inter-individual risk heterogeneity that can be operationalized through risk stratification (50). Identification of higher-risk patients may further facilitate early reinforcement of longer-term risk-factor modification strategies, such as weight management and treatment of sleep-disordered breathing, extending beyond the blanking period (51, 52). The use of a real-world cohort enhances generalizability, and a worked example illustrating nomogram application is provided in Supplementary Table S1.

Several limitations of this study warrant consideration. First, the retrospective, single-center design limits causal inference and generalizability and may be subject to residual confounding despite multivariable adjustment. Although internal bootstrap validation demonstrated stable discrimination with minimal overfitting, external validation across different populations, centers, and ablation strategies is required. Second, early recurrence was defined using a three-month blanking period in accordance with conventional practice; however, evolving guideline recommendations may refine this temporal framework, and future studies should evaluate alternative definitions and extend risk prediction to longer-term outcomes. Third, all patients received standardized short-term amiodarone therapy; given its long half-life and delayed pharmacodynamic effect, early recurrence may occur before full therapeutic exposure, and uniform post-ablation antiarrhythmic protocols may limit external applicability. In addition, reliance on prior clinical diagnoses of obstructive sleep apnea syndrome may have resulted in under-recognition of undiagnosed cases, potentially attenuating observed associations. The absence of detailed procedural variables, such as lesion set completeness and operator experience, may also have precluded identification of additional determinants of early recurrence. Finally, left atrial diameter was used as a surrogate of atrial structure, whereas volumetric and three-dimensional echocardiographic measures may provide incremental prognostic value. Future prospective, multicenter studies incorporating standardized volumetric imaging, procedural metrics, larger cohorts, and longer follow-up are warranted to validate and refine the proposed nomogram and to determine whether early risk stratification can translate into improved outcomes through targeted risk-factor optimization, particularly weight management and treatment of sleep-disordered breathing.

## 5 Conclusions

Elevated BMI, enlarged LAD, increased BNP, persistent AF, and OSAS emerged as independent predictors of early recurrence following radiofrequency ablation. A nomogram integrating these factors demonstrated robust discrimination and calibration, offering a practical tool for individualized risk stratification. However, external validation in larger, multicenter cohorts is required to confirm these findings.

## Data Availability

The raw data supporting the conclusions of this article will be made available by the authors, without undue reservation.
