# Supplementary material for: Analysis of risk factors for early recurrence after radiofrequency ablation in patients with atrial fibrillation and construction of a nomogram predictive model
Source: Front Cardiovasc Med. 2026 Feb 17;13:1659637. doi: 10.3389/fcvm.2026.1659637 (PMC12953515; doi:10.3389/fcvm.2026.1659637)
Supplement: Supplementary file 1 [file Table1.docx]

**Supplementary Table 1. Example of Nomogram Application for Individual Risk Prediction**

To illustrate the clinical application of the nomogram, we present a representative patient example and the corresponding scoring process.

Example Patient

A 65-year-old male patient underwent first-time radiofrequency catheter ablation for atrial fibrillation. His clinical characteristics were as follows:

1. Body mass index (BMI): 27.5 kg/m²
2. Left atrial diameter (LAD): 44 mm
3. B-type natriuretic peptide (BNP): 620 ng/L
4. Type of atrial fibrillation: Paroxysmal AF
5. Obstructive sleep apnea syndrome (OSAS): Absent

| Predictor | Patient value | Nomogram points* |
| --- | --- | --- |
| Body mass index (kg/m²) | 27.5 | 100 |
| Left atrial diameter (mm) | 44 | 80 |
| B-type natriuretic peptide (ng/L) | 620 | 54 |
| Atrial fibrillation type | Paroxysmal | 0 |
| Obstructive sleep apnea syndrome | No | 0 |
| Total score | — | 234 |
| Predicted probability of early recurrence | — | ≈ 0.87 |

*Points were approximated based on the nomogram scale.

Interpretation: Based on the nomogram, this patient accumulated a total score of 234 points, which corresponds to an estimated 87% probability of early atrial fibrillation recurrence within three months after radiofrequency ablation. Despite having paroxysmal atrial fibrillation and no history of obstructive sleep apnea syndrome, the markedly elevated body mass index, enlarged left atrial diameter, and increased B-type natriuretic peptide level contributed substantially to the overall risk score. This example highlights how the nomogram integrates multiple risk factors and allows individualized risk stratification, even in patients without traditionally high-risk arrhythmia subtypes or comorbidities.
